# Demographic and clinical characteristics of patients with low back pain in primary and secondary care settings in Southern Denmark

**DOI:** 10.1080/02813432.2023.2196548

**Published:** 2023-05-08

**Authors:** Anders Hansen, Lars Morsø, Mette Jensen Stochkendahl, Merethe Kirstine Kousgaard Andersen, Berit Schiøttz-Christensen, Simon Dyrløv Madsen, Anders Munck, Jesper Lykkegaard

**Affiliations:** a Spine Centre of Southern Denmark, Lillebaelt Hospital, Middelfart, Denmark; b Department of Regional Health Research, University of Southern Denmark, Odense, Denmark; c OPEN, Open Patient Data Explorative Network, Odense University Hospital, Region of Southern Denmark, Odense, Denmark; d Research Unit OPEN, Department of Clinical Research, University of Southern Denmark, Odense, Denmark; e Chiropractic Knowledge Hub, Odense, Denmark; f Department of Sports Science and Clinical Biomechanics, University of Southern Denmark, Odense, Denmark; g Research Unit of General Practice, Department of Public Health, University of Southern Denmark, Odense, Denmark

**Keywords:** Low back pain, primary health care, general practitioners, chiropractic, physical therapy speciality

## Abstract

**Objective:**

To describe and compare the demographic and clinical characteristics of patients with acute or chronic low back pain across all health care settings treating this condition.

**Design and setting:** Concurrent prospective survey registration of all consecutive consultations regarding low back pain at general practitioners, chiropractors, physiotherapists, and the secondary care spine centre in Southern Denmark.

**Subjects:**

Patients ≥16 years of age with low back pain.

**Main outcome measure:**

Demographic characteristics, symptoms, and clinical findings were registered and descriptively analysed. Pearson’s chi-square tested differences between the populations in the four settings. Multiple logistic regression assessed the odds of consulting specific settings, and *t*-test assessed differences between patients attending for a first and later consultation.

**Results:**

Thirty-six general practitioners, 44 chiropractors, 74 physiotherapists, and 35 secondary care Spine Centre personnel provided information on 5645 consultations, including 1462 first-visit consultations. The patients differed significantly across the settings. Patients at the Spine Centre had the most severe symptoms and signs and were most often on sick leave. Compared to the other populations, the chiropractor population was younger, whereas the physiotherapist population was older, more often females, and had prolonged symptoms. In general practice, first-time consultations were with milder cases while patients who attended for a second or later consultation had the worst symptoms, findings, and risk of sick leave compared to the other primary care settings.

**Conclusion:**

The demographic and clinical characteristics of patients with low back pain differ considerably across the health care settings treating them.

**KEY POINTS**
- The study describes the symptoms and clinical findings of patients with low back pain consulting the Danish health care system in all its settings.
- Patients with chiropractors were youngest, while those with physiotherapists were the oldest and most frequently female.
- First consultations in general practice were generally with the least symptomatic patients while those returning for a subsequent consultation had more severe disease including more sick leave compared to patients in the other primary care settings.
- Our findings call for caution when generalizing between health care settings for patients with low back pain.

## Introduction

Low back pain (LBP) is a widespread and burdensome condition [1] and a significant reason for seeking health care [2]. In Denmark patients with LBP are predominantly treated in four settings, in primary care by general practitioners (GPs), chiropractors, and physiotherapists and in secondary care by the regional spine centre.

The same national guidelines for the management of LBP apply to all four settings, recommending a combination of education, self-care advice, exercise- and manual therapy, generally advising against pharmacological pain treatment, and restricting the advice for imaging to patients with trauma, paresis, suspected malignancy, or prolonged severe pain [3]. Neither the patients’ clinical characteristics nor the use of screening instruments have been proven successful in guiding tailored treatment or predicting the course of LBP [4–7].

Comparable data from all four settings are needed to overview the patient population with low back pain in Denmark. Understanding the differences between the populations in the four setting can be used to interpret differences in management and improve coordination and collaborations across the settings. This study aims to describe and compare the clinical characteristics and demographics of patients with acute or chronic LBP across all the main providers of health care for this condition in Denmark.

## Methods

### Design

We conducted a concurrent prospective survey of all consecutive consultations across the four settings. According to Danish legislation, the authorised legal department at the University of Southern Denmark approved the study (ID # 11.220).

### Context

Denmark has 5.8 million citizens. The five governmental regions manage the hospitals and all publicly licensed primary care providers, including GPs, chiropractors, and physiotherapists.

GPs are in general the first point of contact to the Danish healthcare system. All citizens can consult a GP free of charge with any type of healthcare problem [8]. More than 98% of all citizens are listed with a general practice. The GPs can refer patients to specialists, including the other settings in this study.

Chiropractors diagnose, treat, and prevent mechanical disorders of the musculoskeletal system [9]. Patients at chiropractors receive a small regional subsidy, not requiring a referral from the GP. The rest of the cost is paid by the patient or covered by private health insurance, held in 2021 by 2.3 mill. Danish citizens [10].

Physiotherapists aim to help patients develop, maintain and restore maximum movement and functional ability throughout their lifespan [11]. Treatment costs are moderately subsidised by the region provided that the patient is referred by a GP. The rest is paid by the patient or covered by private health insurance, that usually only covers the patient part after the reduction of the subsidy.

GPs and chiropractors can order Magnetic Resonance Imaging of the spine. They can also refer the patient to the Spine Centre which is a public secondary care outpatient facility providing fully tax-paid specialized diagnostics and treatment. It comprises a surgical and a medical department. The personnel include medical doctors, chiropractors, physiotherapists, and nurses.

### Procedures

We invited all licenced GPs, chiropractors, and physiotherapists listed in the governmental Region of Southern Denmark and all medical personnel at the Spine Centre to participate in the study. The registration period was 1–14 November 2019 for chiropractors, physiotherapists, and the Spine Centre, while GPs had a registration period of four weeks due to a lower estimated rate of LBP consultations. The settings were instructed to consecutively register all LBP face-to-face consultations, except for home visits. The registration procedure followed the Audit Project Odense (APO) method, a prospective self-reporting survey used in many healthcare settings worldwide [12,13]. Following a one-page instruction, clinicians were told to fill in a registration chart during or immediately after each consultation, spending an estimate of two minutes per registration. The authors designed the registration chart with stakeholders representing the four settings. Revision based on a pilot test performed by three to five representatives from each setting improved the acceptability and utility of the registration procedures and specified minor corrections.

### Data

Along with the survey registration, each clinician provided information on their age, sex, and years of practice. The APO survey registration chart included between 45 and 47 items per setting. Twenty-two identical variables provided information on patient characteristics, symptomatology, and consultation findings. The remaining variables on management varied depending on the setting. Only identical variables are included in this study. Patient characteristics included age, sex, consultation number, duration of symptoms in the current LBP episode, whether the patient had none, few, or more prior LBP episodes, and which of the other health settings the patient had consulted during the present episode of LBP. Furthermore, the chart included information on symptomatology and findings, including radiculopathy below knee level, multisite pain (>2 painful body regions), disability including sleep disturbances, physical impairments, emotional distress, sick leave, if a neurological examination was made if abnormal spine-related neurologic findings were found, and if the symptoms and signs indicated nerve root compression. The general practitioner registration chart can be found in the e-supplementary materials section.

### Analyses

Descriptive statistics and variance measures were used to present differences across the populations of patients with LBP who consulted the settings. The *χ*^2^ test was used for categorical variables, and the Kruskal–Wallis test was used for non-normally distributed data. Multivariate logistic regressions with odds ratios (ORs) and 95% confidence intervals (CI) were used to examine if specific parameters were associated with the recording healthcare setting. *T*-tests were performed to assess differences between patients attending for the first or second or later consultation in the respective settings. Missing data were handled by pairwise deletion, and a statistical significance level of 5% was used. All analyses were performed using Stata IC version 17 software (StataCorp LP, College Station, Texas).

## Results

In this study, 189 clinicians participated, yielding a 4%, 64%, 10%, and 63% participation rate for GP, chiropractors, physiotherapists, and Spine Centre personnel, respectively. There were no statistically significant differences in age, sex, or years in practice across clinicians from the settings. However, the number of registered consultations per clinician varied widely (1–138 consultations registered). Data are not shown.

A total of 5645 consultations were included in the analysis. The mean age of the study population was 53 years, ranging from 18 to 96 years, with chiropractors having the lowest mean age (51 years) and physiotherapists having the highest mean age (57 years). The gender composition was 54% female, with chiropractors having the lowest percentage of females (49%) and physiotherapists having the highest percentage (60%).

The clinicians registered 1462 (26%) as first-time consultations. In 63% of the first-time consultations, the patient had previously experienced multiple LBP episodes (Table 1). In 1267 (24%) consultations, the patient presented with radiating symptoms below knee level; in 1649 (30%) consultations, the patient had pain in multiple body regions. Disability was highly prevalent in the cohort. In 5076 (90%) consultations, the patient was in some way functionally affected by the LBP. Reported disabilities were composed of physical impairments (4442 consultations; 78%), sleep disturbance (2159 consultations; 38%), and emotional distress (1424 consultations; 25%), and in 727 (13%) of the consultations, the patient was on sick leave. Of the 5.645 consultations, 3.886 (69%) included a neurological examination, and an abnormal spine-related neurological finding was registered in 695 (12%).

**Table 1.** Characteristics of patients with low back pain in different healthcare settings.

Variable	Group	Total data	GP	DC	PT	SC	Missings	*p* diff
		*n* (%)						
Consultations		5645 (100)	273	2785	1733	854		<.01
Age	16–34	834 (15)	36	502	163	131		
	35–49	1501 (27)	68	842	368	223		
	50–65	1933 (34)	99	932	625	277		
	>65	1304 (23)	68	467	574	195	73 (1)	<.01
Gender	Female	3012 (53)	155	1362	1037	458		
	Male	2608 (46)	118	1421	686	383	25 (1)	<.01
Consultation #	1st	1462 (26)	149	683	308	322		
	2–4	2258 (40)	71	1212	577	398		
	5–9	925 (16)	6	525	332	62		
	10–14	242 (4)	3	120	112	7		
	≥15	661 (12)	31	238	386	6	97 (2)	<.01
Duration in weeks	0–1	996 (18)	63	804	126	3		
	2–4	1149 (20)	58	823	23	33		
	5–8	424 (8)	9	243	150	22		
	>8	2745 (49)	99	878	1162	606	331 (6)	<.01
Previous LBP episodes	None or few	1796 (32)	121	901	536	238		
	More	3664 (65)	145	1848	1178	493	97 (2)	<.01
Also treated by	GP	–	–	776	1413	–*		
	DC	625 (11)	53	–	275	297		
	PT	1138 (20)	105	542		491		
	SC/hospital	811 (14)	48	210	391	162		
	None	2066 (37)	102	1713	208	43	n/a	<.01

GP: General practitioner; DC: Chiropractor; PT; Physiotherapist; SC: Spine Centre.

*Not reported as all patients were expected to have consulted a GP before the SC visit.

There were statistically significant differences across the populations in the four settings regarding all the measures (Table 2). The regression analysis demonstrated that patients with radiculopathy below knee level, physical impairments, emotional distress, sick leave, or abnormal neurology had higher odds of consulting the Spine Centre than the three primary care settings. In the primary care settings, patients with radiating pain below knee level had higher odds of consulting a general practitioner than a chiropractor or a physiotherapist. Patients with multisite pain, sick leave, and abnormal neurology had higher odds of consulting a GP than a chiropractor (Table 3).

**Table 2.** Symptoms and findings of patients with low back pain at different healthcare settings.

Variable	Total data	GP	DC	PT	SC	Missing	*p* diff*
	*n* (%)					*n* (%)	
Symptoms							
Radiating pain							
Without or above knee level	4044 (72)	188	2270	1271	315		
Beneath knee level	1267 (22)	68	406	389	404	334 (6)	<.01
Multisite pain	1649 (29)	80	572	741	256	109 (2)	<.01
Functioning							
Sleep	2159 (38)	112)	959	689	399		<.01
Emotionally	1424 (25)	63	514	502	345		<.01
Physically	4442 (79)	206	2168	1364	704		<.01
Sick-listed	727 (13)	47	301	184	195		<.01
None	569 (10)	30	357	157	25	109 (2)	<.01
Neurology							
Normal	3191 (57)	192	1663	1016	320		<.01
Abnormal	695 (12)	46	228	242	179		<.01
Untested	1533 (27)	33	868	539	93	137 (2)	<.01
Suspected nerve root compression	578 (10)	41	222	169	146	137 (2)	<.01

GP: General practitioner; DC: Chiropractor; PT: Physiotherapist; SC: Spine Centre.

*p-differences are calculated by use of chi-square tests.

**Table 3.** Adjusted comparison of symptoms and findings of patients with low back pain at different healthcare settings.

Variable	GP	DC	PT	SC
	Ref	OR (CI 95%)**	OR (CI 95%)**	OR (CI 95%)**
Symptoms:				
Radiating pain beneath knee level	1	0.47 (0.34;0.65)*	0.59 (0.42;0.83)**	2.62 (1.82;3.78)*
Multi-site pain	1	0.65 (0.47;0.89)*	1.31 (0.95;1.82)	0.99 (0.70;1.43)
Functioning				
Sleep	1	0.80 (0.60;1.05)	0.09 (0.74;1.32)	1.31 (0.95;1.81)
Emotionally	1	0.78 (0.56;1.01)	1.22 (0.86;1.71)	2.03 (1.40;2.92)*
Physically	1	1.20 (0.86;1.66)	1.44 (0.99;1.96)	3.42 (2.22;5.28)*
Sick-listed	1	0.44 (0.30;0.65)*	0.70 (0.47;1.05)	2.12 (1.38;3.26)*
None	1	1.05 (0.67;1.62)	0.74 (0.47;1.18)	0.30 (0.16;0.56)*
Findings:				
Abnormal Neurology***	1	0.53 (0.36;0.78)*	0.77 (0.52;1.14)	2.19 (1.42;3.36)*
Suspected nerve root compression***	1	0.75 (0.44;1.23)	0.59 (0.34;1.02)	0.84 (0.47;1.54)

GP: General practitioner; DC: Chiropractor; PT: Physiotherapist; SC: Spine Centre.

*p < 0.05, **Odds ratios are adjusted for age, gender, consultation number, and symptom duration, ***The analysis excludes patients without a neurological examination.

**Figure 1.**
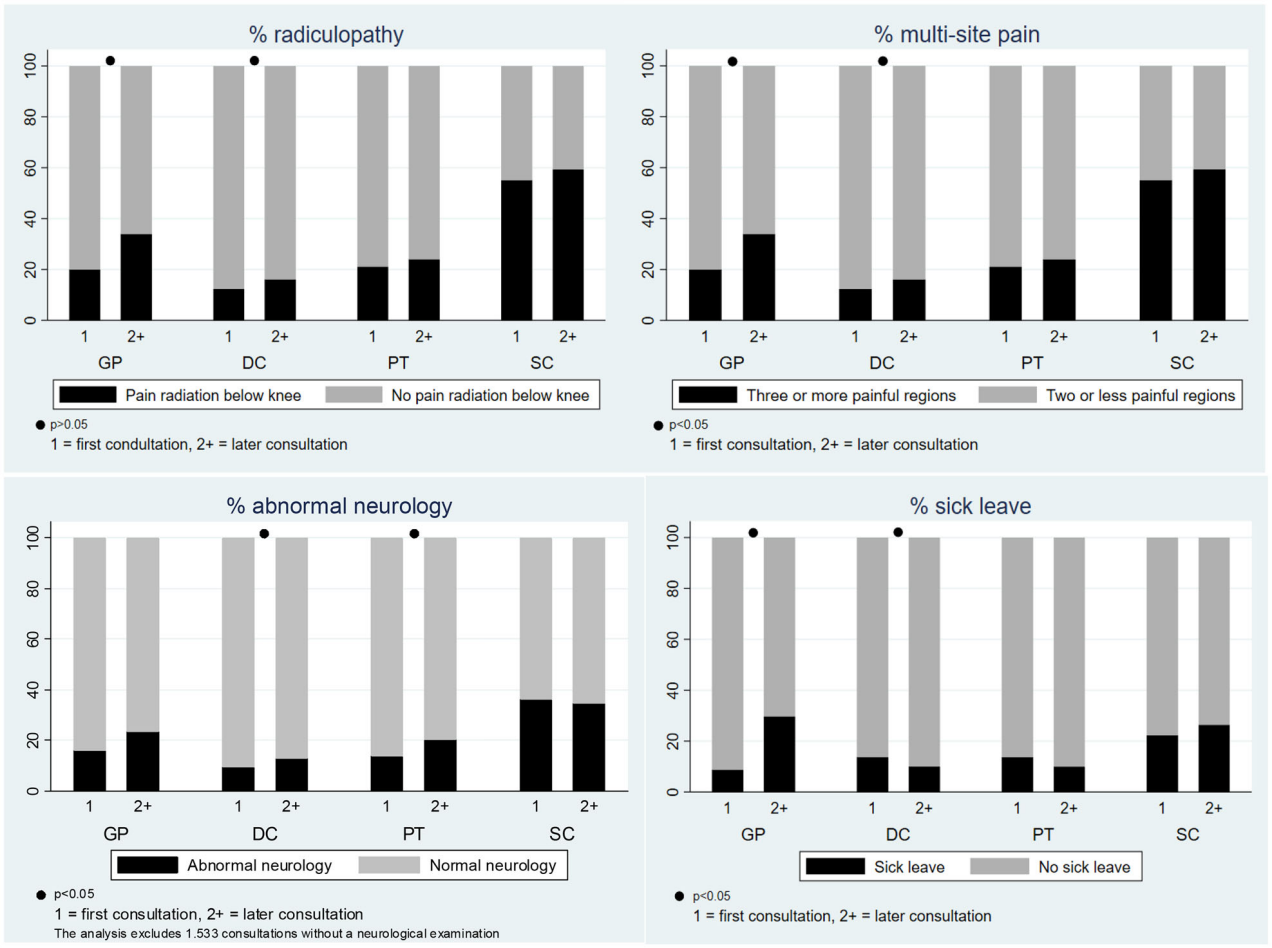
Severity measures of patients with low back pain who consult general practitioners, chiropractors, physiotherapists, and the spine centre at 1st and 2nd or later visit. Graph combined of four graphs, each with the groups of consultation numbers on the x-axis and showing patients with the four health professions. Y-axis 1A: Percentage radiculopathy, 1B: Percentage multisite pain. 1C: Percentage of abnormal neurology, and 1D: Percentage of sick leave. GP: General practitioner; DC: Chiropractor; PT: Physiotherapist; SC: Spine Centre.

There were statistically significant differences between patients attending first-time and second or later consultations across the three primary care settings. Patients who consulted the GP for a second or later consultation had significantly higher proportions of radiculopathy (*p* < .01), multisite pain (*p* < .01), and sick leave (*p* < .01) than patients at first-time consultations. Higher severity measures of multisite pain (*p* < .02), radiculopathy (*p* < .02), and abnormal neurology (*p* < .04) were also present in the chiropractor population at later consultations compared to the first. However, fewer patients were on sick leave at chiropractors when returning for a second or later consultation (*p* < .02). The pattern for patients consulting physiotherapists for a first compared to second or later consultation was similar to chiropractors, except that only the increase in abnormal neurology reached statistical significance (Figure 1). Only minor changes were observed at the Spine Centre between the first- and second or later consultations.

## Discussion

Our study found significant differences in the LBP patient populations across the four main healthcare settings constituting the first- and second-line treatment of patients with LBP in Denmark. Patients with the most severe and extended symptom duration are treated in secondary care. Patients who consult chiropractors are generally younger, have less severe symptoms and are treated earlier in the LBP episodes than in the other settings. Patients who consult physiotherapists are older, more often females, have more prolonged symptoms, and have more consultations per patient than in other settings. For patients who consult the GP, the severity of symptoms and proportion on sick leave are higher at second or later than first-time consultations. This pattern is not as apparent in the other settings, where sick leave is lower at second or later consultations. A plausible explanation for this pattern may be that GPs often starts with a wait-and-see approach informing the patient to return in case of worsening or lack of improvement. In comparison, chiropractors and physiotherapists often plan a course of treatment running one or more weeks. Therefore, returning to the GP usually implies a more severe disease, whereas returns to chiropractors or physiotherapists are planned to happen even if the condition improves. Some returns of patients with LBP to the GP may be of the patient on opioids who must show up for prescription renewal or of patients in need of declaration regarding sick leave.

The population differences across the three primary care settings are likely to be caused by multiple factors, such as the out-of-pocket expense related to chiropractic and physiotherapy visits, supply, and demand of treatment types, waiting time, and advertisement. Appointments with the GP are fully tax paid, and no referral is required. Chiropractors have the highest out-of-pocket cost (∼80%). That for physiotherapists is lower (∼60%) but requires a GP referral. Cost considerations and lower proportion with employer-paid health insurance may, to some extent, explain why older patients tend to seek GPs and physiotherapists, whereas younger male patients tend to consult chiropractors. Older people usually have many consultations with the GP for other reasons and may therefore be more likely to use the GP for LBP. The distribution of patients across the healthcare settings may also be influenced by numerous patient factors not included in this survey, such as comorbidity, medication, previous health history, cultural beliefs, and illness perception.

### Strength and limitations

The study used the well-established APO method to survey consecutive cases. Before initiating the registration process, the feasibility of the survey chart was thoroughly evaluated to ensure that it was well-designed and could be effectively implemented. A key strength of the study was its comprehensive sampling strategy, which included patients from Denmark’s four main healthcare settings that treat patients with LBP. The large sample size allowed a description of various parameters, resulting in a comprehensive overview of the demography and clinical characteristics of patients with LBP in Denmark. The data saturation was high, with limited missing information per registration, increasing the validity of the study’s findings. Researchers from all four settings conducted the data collection, analysis, and interpretation of the findings, and the study was mutually funded by all four settings reducing the risk of biased comparison of the settings.

Only a small proportion of all GPs in the region (4%) participated in the study. Since participation was voluntary, they may be a selected group, e.g. with a particular interest in LBP. However, especially the characteristics of first-time consulting patients are not likely to be highly affected by the GPs interest in LBP. Later consultations are more likely to be subject to selection bias. Nevertheless, the presented differences in patient characteristics between the four settings are more likely to relate to the settings than to be caused by biased sampling.

The sampling over a few weeks favours the inclusion of frequently attending patients, such as patients treated with opioids in general practice or having longer treatment courses at chiropractors or physiotherapists. On the other hand, our findings reflect the patients most frequently met in the everyday clinic.

In Denmark, other healthcare professions care for LBP than those included in this study e.g. osteopaths. However, they only care for a very small proportion of the patients and are not formally integrated with the healthcare system.

The APO-method does not allow the identification and follow-up of individual patients, and we do not know if a patient appears more than once in the data. Thus, we cannot identify what happens throughout treatment courses. For example, approximately 1/3 of the consultations did not include a neurological examination, and we do not know if a patient was never neurologically examined or if the examination was performed in other consultations with the patient than the one registered. It is likely that neurological examination is performed more often in first-time consultations and considered less clinically meaningful in follow-up consultations with neurologically unaffected patients.

The study did not register information on how the treatment costs were covered e.g. the use of private health insurance. This would have been interesting for our interpretation of the differences between the patients with chiropractors and physiotherapists.

Finally, the clinicians in the four settings have different roles and access to the patient’s health records and may have emphasised some information over others. For example, GPs may be more likely to record pain medication and sick leave, whereas physiotherapists may be more aware of physical impairments and functioning levels.

### Findings in relation to other studies

The mean age of our populations was 50–60 years, and 54% were women. Disability was present in 90% of our patients, frequently recurrent LBP episodes were reported in 66%, and over 10% had sick leave. With regard to LBP populations, these findings are consistent [14]. Previous research has identified gender and disability as factors associated with care-seeking for LBP [15]. Our results are consistent with data from the Danish Chiropractic Low Back Pain Cohort, which included 2848 patients [16]. A study of 1250 patients with LBP who consulted GPs and chiropractors also found that patients who sought care for chiropractors were slightly younger and had milder symptoms than those consulted with GPs [17]. This was also found in Canada [18].

### Meaning of the study

By including consecutive consultations from all the main settings that treat patients with LBP in Denmark, our study describes the essential characteristics of patients who consult the Danish health system with LBP. To our knowledge, no prior studies have reported on the overall LBP patient population attending a health system in all its settings.

Each setting is aware of the characteristics of its patients but may unconsciously assume that patients in the other settings are similar, leading to misunderstandings. Potential misunderstandings caused by this erroneous assumption may be avoided if considering our findings. Thereby, awareness of our findings may facilitate further collaboration and coordination across the four settings.

## Conclusion

We have described the demographic and clinical characteristics of people seeking health care for LBP in Southern Denmark. Age, gender, and clinical characteristics differ across the settings. This should be considered when comparing and interpreting findings from different settings.

## Supplementary Material

Supplemental Material
