# Supplementary material for: Demographic and clinical characteristics of patients with low back pain in primary and secondary care settings in Southern Denmark
Source: Scand J Prim Health Care. 2023 May 8;41(2):152–9. doi: 10.1080/02813432.2023.2196548 (PMC10193863; doi:10.1080/02813432.2023.2196548)
Supplement: Supplemental Material [file IPRI_A_2196548_SM8258.pdf]

ID:

# Low Back Pain (LBP) – Registration chart - GPs

Date: ____________

## Patient characteristics

| | Age (years) | Sex | | Consultation | | Prior LBP episodes | | During this LBP episode, incl. if referred to | | | | | Present symptoms | | Characteristics | | | | | | Clinical findings and assessment | | | | |
|---|---|---|---|---|---|---|---|---|---|---|---|---|---|---|---|---|---|---|---|---|---|---|---|---|---|
| | | | | Type number | | 1 X | | min 1 X | | | | | 1 X | | min 1 X | | | | | | 1 – 3 X | | | | |
| | | | | Consultation number during the LBP episode | No. weeks with symptoms during the LBP episode | Several disabling episodes with LBP | None or few episodes with LBP | Chiropractor | Physiotherapist | Spine Centre/other hospital dept/surgery | MRI scan | None of the above | LBP without radiation or maximally to knee-level | LBP with radiation beneath knee-level | Pain in two or more regions other than the LBP | Poor sleep | Physically disabled by the LBP | Emotionally affected by the LBP | On sick leave | None of the above | Abnormal neurology | Normal neurology | Neurological examination not performed | Suspected nerve root compression | Suspected severe pathology |
| | | M | F | 1 | 2 | 3 | 4 | 5 | 6 | 7 | 8 | 9 | 10 | 11 | 12 | 13 | 14 | 15 | 16 | 17 | 18 | 19 | 20 | 21 | 22 |
| 1 | | | | | | | | | | | | | | | | | | | | | | | | | |
| 2 | | | | | | | | | | | | | | | | | | | | | | | | | |
| 3 | | | | | | | | | | | | | | | | | | | | | | | | | |
| 4 | | | | | | | | | | | | | | | | | | | | | | | | | |
| 5 | | | | | | | | | | | | | | | | | | | | | | | | | |
| 6 | | | | | | | | | | | | | | | | | | | | | | | | | |
| 7 | | | | | | | | | | | | | | | | | | | | | | | | | |
| 8 | | | | | | | | | | | | | | | | | | | | | | | | | |
| 9 | | | | | | | | | | | | | | | | | | | | | | | | | |
| 10 | | | | | | | | | | | | | | | | | | | | | | | | | |

## Today's consultation

| | Actions and prescriptions today | | | | | | | | | | Referrals sent or recommended today | | | | | | | | Aims of today's consultation | | | | | | |
|---|---|---|---|---|---|---|---|---|---|---|---|---|---|---|---|---|---|---|---|---|---|---|---|---|---|
| | min 1 X | | | | | | | | | | min 1 X | | | | | | | | min 1 X | | | | | | |
| | Advice given incl. on self-management | Informed of LBP symptoms and prognosis | Instructed to do specific exercises | Manually treated | Acupuncture or injektion | NSAID | Gabapentin/pregabalin/tricyclic antidepressant | Opioid | New consultation booked regarding LBP | None of the above | Physiotherapist | Chiropractor | Spine Centre | Acute admission or cancer fast track | Lumbar MRI | Municipality LBP management | Extended LBP examination at PT or DC | None of the above | Find the cause of the LBP | Reassure the patient | Meet the patient's desires | Relieve pain | Promote the patient's self-management | Issue medical certification | None of the above |
| | 23 | 24 | 25 | 26 | 27 | 28 | 29 | 30 | 31 | 32 | 33 | 34 | 35 | 36 | 37 | 38 | 39 | 40 | 41 | 42 | 43 | 44 | 45 | 46 | 47 |
| 1 | | | | | | | | | | | | | | | | | | | | | | | | | |
| 2 | | | | | | | | | | | | | | | | | | | | | | | | | |
| 3 | | | | | | | | | | | | | | | | | | | | | | | | | |
| 4 | | | | | | | | | | | | | | | | | | | | | | | | | |
| 5 | | | | | | | | | | | | | | | | | | | | | | | | | |
| 6 | | | | | | | | | | | | | | | | | | | | | | | | | |
| 7 | | | | | | | | | | | | | | | | | | | | | | | | | |
| 8 | | | | | | | | | | | | | | | | | | | | | | | | | |
| 9 | | | | | | | | | | | | | | | | | | | | | | | | | |
| 10 | | | | | | | | | | | | | | | | | | | | | | | | | |

®Copyright: Audit Projekt Odense, J.B. Winsløws Vej 9A, 1. 5000 Odense C
